# Metabolic Profile of Indigo Naturalis in Rat Plasma, Bile, Urine, and Feces by UPLC–Q‐TOF MS Method

**DOI:** 10.1155/jamc/8592761

**Published:** 2026-10-27

**Authors:** Guozhe Zhang, Jianting Liu, Dan Yuan, Yiyu Qin

**Affiliations:** ^1^ Department of Translational Medicine Jiangsu Medical College 283 South of Republic Road Yancheng 224005 China; ^2^ Department of Traditional Chinese Medicine Shenyang Pharmaceutical University 103 Wenhua Road Shenyang 110016 China syphu.edu.cn

**Keywords:** indigo naturalis, metabolic pathways, metabolites, UPLC–Q-TOF MS

## Abstract

Indigo naturalis (IN, Qing‐Dai) is a well‐established traditional Chinese medicine (TCM) with extensive biological activities, particularly in the clinical management of ulcerative colitis (UC) and psoriasis. However, the comprehensive in vivo metabolic behavior of IN extract remains inadequately characterized. This research employed ultra‐performance liquid chromatography/quadrupole time‐of‐flight mass spectrometry (UPLC–Q‐TOF MS) methodology to comprehensively examine the metabolic characteristics of IN in rat models. Biological samples, including plasma, bile, urine, and feces, were obtained from rats following single oral administration of IN extract. Sample preparation techniques involved protein precipitation, solid‐phase extraction, and ultrasonic extraction for processing plasma, bile, urine, and feces specimens, respectively. Through precise mass measurement and fragment ion analysis, we identified 24 prototype compounds and 37 metabolites and clarified the transformation relationships between prototypes and their metabolites. The biotransformation pathways of IN components in rats primarily involved N‐ or O‐glucuronidation, N‐ or O‐sulfation, hydroxylation, dehydroxylation, demethylation, demethoxylation, decarbonylation, and reduction reactions. Our findings demonstrate that N‐glucuronidation and N‐sulfation represent the predominant metabolic reactions. This investigation marks the first systematic report on metabolite identification and metabolic pathway speculation of major constituents following oral administration of IN extract, providing a scientific foundation for understanding its in vivo processes.

## 1. Introduction

Indigo naturalis (IN, Qing‐Dai), a traditional Chinese medicine (TCM) with heat‐clearing and detoxifying properties, has been utilized in China for centuries. This dark‐blue powder, lump, or granular substance originates from the leaves and stems of *Baphicacanthus cusia* (Nees) Bremek., *Polygonum tinctorium* Ait., or *Isatis indigotica* Fort. Its complex chemical composition mainly includes indole alkaloids (such as indigo and indirubin), quinazolinone compounds (including 4(3H)‐quinazolinone and quinazoline‐2,4(1H,3H)‐dione), and terpenoids (such as *α*‐pinene and loliolide), along with other constituents. A comprehensive review by Sun et al. [1] summarized 63 chemical compounds isolated from IN, including indole alkaloids, terpenoids, organic acids, steroids, and nucleosides, and noted that systematic metabolic profiling of IN extract in vivo remains limited.

Contemporary research has revealed that IN exhibits broad‐spectrum biological activities, including anti‐inflammatory [2], antioxidant [3], antimicrobial [4, 5], and antitumor [6–8] effects. In clinical practice, IN is widely employed for treating ulcerative colitis (UC) [9–12], psoriasis [13–15], and various dermatological conditions [16].

As TCMs are predominantly administered orally, comprehensive evaluation of drug absorption, distribution, metabolism, and excretion processes holds significant importance. IN demonstrates poor water solubility, yet oral formulations remain common in clinical practice, particularly for UC treatment and antitumor applications. Previous reports indicate that IN may induce adverse effects, including headache, gastrointestinal symptoms, mild hepatic impairment [17, 18], and pulmonary arterial hypertension [19]. Adachi and colleagues [20] discovered that IN could ameliorate oxazolone–induced dermatitis while exacerbating colitis through modulation of gut microbiota composition, highlighting the necessity for detailed investigation of its in vivo metabolic behavior.

Although metabolic studies of individual components such as isatin, meisoindigo, indirubin, deoxyvasicinone, and tryptanthrin have been preliminarily conducted [21–26], the comprehensive metabolic profile of IN remains inadequately characterized. Tang et al. [27] conducted a human pharmacokinetic study of IN‐derived indole alkaloids, revealing that tryptanthrin, despite its low abundance in IN (0.3%), exhibited the highest systemic exposure and was the major contributor to aryl hydrocarbon receptor (AhR) agonistic activity in the first 6 h after oral ingestion, while indirubin contributed to later AhR activity. This highlights the importance of understanding the metabolic fate of individual IN constituents. This study utilized ultra‐performance liquid chromatography/quadrupole time‐of‐flight mass spectrometry (UPLC–Q‐TOF MS) technology to thoroughly analyze the metabolic characteristics in rat biological samples following oral administration of IN, elucidating its metabolic pathways in rats and speculating on transformation relationships between prototype components and metabolites, thereby providing a theoretical foundation for understanding IN *in vivo* behavior and potential adverse reactions.

## 2. Materials and Methods

### 2.1. Chemicals and Reagents

IN was supplied by Beijing “Tong Ren Tang” Pharmaceutical Co., Ltd. Six reference standards (purity > 98%) comprising quinazoline‐2,4(1H,3H)‐dione, 4(3H)‐quinazolinone, isatin, tryptanthrin, indigo, and indirubin were isolated and purified in our laboratory. Structural identification was performed using ultraviolet, infrared, 1H and 13C nuclear magnetic resonance, and mass spectrometry techniques. HPLC‐grade formic acid and acetonitrile were employed, with ultra‐pure water generated by a Milli‐Q water purification system.

### 2.2. Animal Experiments

A total of 12 male Sprague–Dawley rats were used in this study. For plasma, bile, urine, and feces collection, three rats were assigned to each biological matrix (*n* = 3 per matrix). Animals were acclimatized for one week at 23°C–28°C and 50%–65% humidity and a 12 h light/dark cycle. Rats were fasted for 12 h before metabolic experiments, with free access to water and sugar during sample collection periods.

Due to poor water solubility, IN was suspended in 0.5% sodium carboxymethylcellulose solution at a 2 g/mL concentration, followed by 5‐min sonication to achieve a homogeneous suspension for rat oral administration. Blood samples were collected from the suborbital vein to obtain blank plasma. Following oral gavage of IN at a 20 g/kg body weight dose, blood samples were collected into heparinized polyethylene tubes at 0.25, 0.5, 1, 2, 4, 8, 12, 18, and 24 h time points, immediately centrifuged at 3500 rpm for 10 min at 4°C to separate plasma, and stored at −20°C until analysis. Urine and feces samples were collected over 0–12 h post‐administration at the same 20 g/kg dose. Urine specimens were immediately stored at −20°C, while feces samples were preserved at −20°C after drying. For bile collection, rats were anesthetized by intraperitoneal urethane injection, followed by surgical insertion of a plastic cannula into the bile duct. After collecting blank bile, animals received oral administration of an identical IN dose, with bile samples collected over 0–24 h. Equal volumes of samples from the three rats in each group were pooled prior to analysis to generate representative biological samples for metabolite identification, as previously described [28]. The experimental protocol received approval from the Animal Experimentation Ethics Review Committee of Shenyang Pharmaceutical University.

### 2.3. Preparation of Rat Biological Samples and IN Samples

Plasma: Equal volumes of plasma from each time point were pooled, and 200 μL of mixed plasma was combined with 600 μL methanol, followed by 2‐min vortex mixing. After centrifugation at 10,000 rpm for 10 min to precipitate proteins, supernatants were transferred to fresh tubes and evaporated to dryness under a nitrogen stream at room temperature. The resulting residue was reconstituted with 100 μL methanol–water (70:30, v/v) and centrifuged at 13,000 rpm for 10 min. The 2 μL aliquots were injected into the UPLC–Q‐TOF MS system for analysis.

Urine: Solid‐phase extraction with Bond Elut C18 cartridges (3 mL, 500 mg) was employed for metabolite extraction from urine samples. Cartridges were activated with 2 mL methanol and conditioned with 2 mL distilled water. Pooled urine samples were centrifuged at 3500 rpm for 10 min to remove impurities, and 100 μL supernatants were loaded onto the cartridges. SPE cartridges were washed with 2 mL water to eliminate matrix components, followed by elution with 2 mL methanol to recover metabolites. The methanol fraction was concentrated to dryness under a nitrogen stream. The residue was reconstituted in 200 μL methanol–water (70:30, v/v), centrifuged at 13,000 rpm for 10 min, and 2 μL supernatants were injected for analysis.

Bile: Bile samples underwent similar solid‐phase extraction procedures as urine, with the modification that 100 μL bile sample residue was reconstituted in 100 μL methanol–water (70:30, v/v).

Feces: The ultrasonic extraction method was applied for feces sample preparation. Each 0.1 g feces sample was mixed with 1 mL methanol–water (70:30, v/v). Following 30‐min ultrasonic extraction and centrifugation at 13,000 rpm for 10 min, supernatants were filtered through 0.22 μm membranes, with 2 μL aliquots injected for analysis.

IN sample: Accurately weigh 0.1 g of IN powder into a 10 mL volumetric flask. Add exactly 10 mL of methanol, seal the flask, and weigh again. Sonicate (100 W, 40 kHz) for 30 min, then allow to cool. Reweigh the flask and make up for any lost weight with 100% methanol. Centrifuge at 3500 rpm for 10 min, filter the supernatant through a 0.22 μm membrane, and use the subsequent filtrate as the UPLC–Q‐TOF MS sample.

### 2.4. UPLC and MS Conditions

Chromatographic separation was performed using a Waters ACQUITY HSS C18 column (2.1 × 100 mm, 1.8 μm) maintained at 40°C. The mobile phase consisted of (A) water containing 0.1% formic acid and (B) acetonitrile containing 0.1% formic acid, with gradient elution programmed as follows: 0–2 min, 5%–10% B; 2–4 min, 10%–17% B; 4–4.5 min, 17%–25% B; 4.5–8 min, 25%–40% B; 8–10 min, 40%–50% B; 10–11.5 min, 50%–60% B; 11.5–13 min, 60%–75% B; 13–14 min, 75%–99% B; 14–15 min, 99% B; 15–15.1 min, 99%–5% B; 15.1–16 min, 5% B. Flow rate was maintained at 0.4 mL/min with 2.0 μL injection volume for biological samples.

Metabolite identification of IN in rats was conducted using a Waters Q‐TOF Xevo G2 mass spectrometer equipped with an electrospray ionization source. Leucine‐enkephalin served as the lock mass reference compound, generating [M+H]+ ion (m/z 556.2271) in LockSpray mode at 50 pg/μL concentration. The ESI source operated in positive ionization mode with the capillary voltage set at 3.0 kV. Positive mode was selected because the major IN constituents are nitrogen‐rich indole/quinazolinone alkaloids that protonate readily under ESI and lack strongly acidic functional groups, consistent with established LC–MS methods for these compounds [29]. Cone voltage was maintained at 40 eV. Source and desolvation temperatures were established at 130°C and 450°C, respectively. Cone and nebulization gas flows were set at 50 L/h and 800 L/h. All data acquisition utilized MassLynx NT 4.1 software. MS^E^ mode was employed for data acquisition with a collision energy ramp of 20–40 eV, enabling simultaneous acquisition of molecular ions and fragment ions in single analyses.

Data acquisition and processing were performed using MassLynx NT 4.1 software (Waters, USA). Post‐acquisition data mining for metabolite screening was carried out using MetaboLynx software (Waters), which enabled automated detection of predicted phase I and phase II metabolites based on accurate mass measurements, retention time shifts, and characteristic fragment ions. Potential metabolites were identified by comparing the MS^E^ data of drug‐containing samples with blank controls (plasma, bile, urine, and feces from untreated rats). The following metabolic transformations were mainly considered as screening parameters: glucuronidation (addition of 176 Da), sulfation (addition of 80 Da), hydroxylation (addition of 16 Da), demethylation (loss of 14 Da), reduction (addition of 2 Da), and combinations thereof. Elemental compositions were calculated using the elemental composition tool within MassLynx based on exact mass measurements (mass tolerance ≤ 5 ppm). Proposed structures were further supported by diagnostic fragment ions observed in the high‐energy MS^E^ channel (collision energy ramp of 20–40 eV).

## 3. Results and Discussion

Prior to animal administration, the IN extract was analyzed by UPLC–Q‐TOF MS under the same chromatographic and mass spectrometric conditions described in Section 2.4. The following prototype compounds were identified by comparison of their retention times and mass spectra with the corresponding authentic standards: quinazoline‐2,4(1H,3H)‐dione, 4(3H)‐quinazolinone, isatin, tryptanthrin, indigo, and indirubin. Other prototypes, such as isoindigo and deoxyvasicinone, were tentatively identified based on literature comparison and mass spectrometric fragmentation patterns. The compounds found from IN were shown in Table S1.

Extracted ion chromatograms facilitated identification of prototypes and metabolites in rat biological samples following oral administration of IN, demonstrating superior sensitivity and processability compared to base peak ion chromatograms and total ion chromatograms. Prototype identification involved comparison of retention times and MS/MS data with reference standards or literature data. Investigation of detailed fragmentation pathways of major IN components proved crucial, as metabolite mass fragmentation patterns often resemble those of their prototypes. Structural confirmation of metabolites was achieved by comparing product ion spectra with precursor ions. This process was assisted by Mass Fragment software. Product ion spectra and proposed fragmentation pathways for P3, P4, P5, P13, P16, P18, and P20 are presented in Figure 1.

**FIGURE 1:**
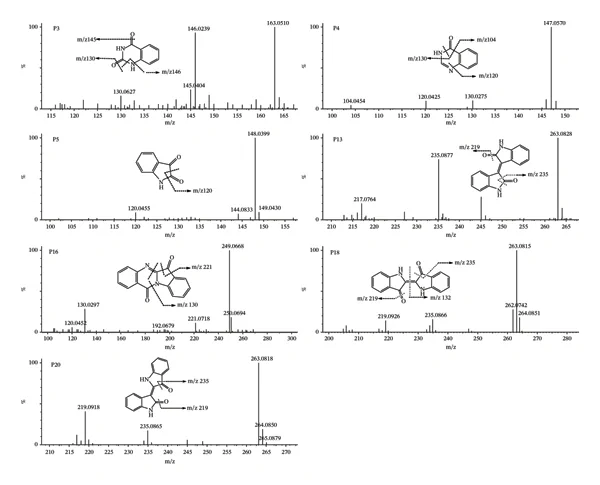
The product ion spectra and proposed fragmentation pathways of P3, P4, P5, P13, P16, P18, and P20.

A novel strategy was implemented for systematic screening and characterization of IN metabolites. Initially, MS^E^ data for both drug‐containing and blank control biological samples were acquired using the developed methodology. Subsequently, Metabolynx software enabled post‐acquisition data mining, rapidly predicting molecular weights and elemental compositions of common and uncommon metabolites based on accurate mass measurements. Finally, metabolite structures were elucidated through integration of accurate mass determination, relevant drug biotransformation knowledge, characteristic fragmentation pathways of IN metabolites, and available literature data.

EICs of prototypes and metabolites in IN‐containing rat biological samples are shown in Figure 2. Tables 1 and 2 summarize retention time, molecular formula, calculated mass, observed mass (molecular/main fragment ions), mass error, metabolite description, and matrix information for the 61 identified components (24 prototypes and 37 metabolites).

**FIGURE 2:**
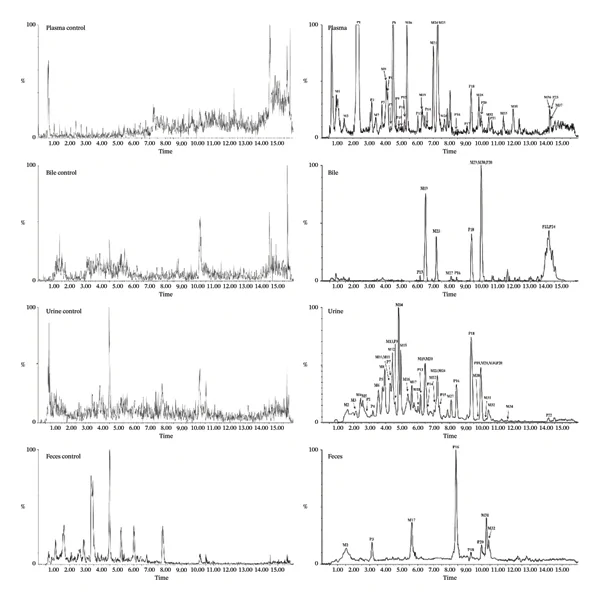
Extracted ion chromatograms (EICs) of the components and metabolites in IN‐containing rat biological samples (plasma, urine, bile, and feces) identified by UPLC‐Q‐TOF MS in positive ion mode.

**TABLE 1:** Prototype compounds (P1–P24) identified in rat biological samples after oral administration of indigo naturalis.

No.	RT (min)	Formula	[M + H]^+^ calculated (Da)^a^	[M + H]^+^ observed (Da)	Mass error^b^ (ppm)	Fragment ions	Identification	Matrix^c^
P1	2.48	C_9_H_7_NO	146.0606	146.0606	0.00	117, 77	Indole‐3‐aldehyde	P
P2	2.80	C_9_H_8_N_2_O	161.0715	161.0713	−1.24	130, 120	2‐Methylquinazolin‐4 (3H)‐one	U
P3	3.18	C_8_H_6_N_2_O_2_	163.0508	163.0520	7.36	146, 145, 120	Quinazoline‐2, 4 (1H,3H)‐dione	P B
P4	3.22	C_8_H_6_N_2_O	147.0558	147.0550	−5.44	130, 120, 104	4 (3H)‐quinazolinone	U
P5	3.81	C_8_H_5_NO_2_	148.0399	148.0400	0.68	120, 91, 92	Isatin	P U
P6	4.20	C_7_H_7_NO_2_	138.0555	138.0568	9.40	120, 92	2‐Amino‐benzoic acid	P
P7	4.33	C_11_H_10_ N_2_O	187.0871	187.0872	0.53	130	Deoxyvasicinone	P U
P8	4.38	C_8_H_7_NO	134.0606	134.0612	4.40	117, 79	Indoxyl	P
P9	4.60	C_10_H_9_NO_2_	176.0712	176.0700	−6.82	159	Indole‐3‐acetic acid	P U
P10	4.81	C_11_H_16_O_3_	197.1178	197.1185	3.50	181, 135	Isololiolide	P
P11	4.98	C_16_H_12_O_6_	301.0712	301.0717	−1.30	285	5,7,4′‐Trihydroxy methoxyflavone	P
P12	5.22	C_11_H_16_O_3_	197.1178	197.1164	−7.10	180, 135	Loliolide	P
P13	6.17	C_16_H_10_N_2_O_2_	263.0821	263.0818	−1.14	235, 219, 190	Isoindigo	P U F
P14	6.53	C_8_H_5_NO_2_	148.0399	148.0400	0.68	130, 120, 105	Phthalimide	P U
P15	7.38	C_18_H_14_N_2_O_3_	307.1083	307.1066	−5.54	289, 263, 261	Phaitanthrin A	U
P16	8.40	C_15_H_8_N_2_O_2_	249.0664	249.0658	−2.41	221, 192, 130	Tryptanthrin	P U B F
P17	9.34	C_18_H_16_ N_2_O	277.1341	277.1351	3.60	248, 219, 160	Halichrome A	P
P18	9.33	C_16_H_10_N_2_O_2_	263.0821	263.0821	0.00	235, 219, 132	Indigo	P U B F
P19	9.91	C_15_H_8_N_2_O_2_	249.0664	249.0663	0.40	221, 192, 166	7‐Oxo‐7H‐benzopyrano[2,3‐c] cinnoline	U
P20	9.95	C_16_H_10_N_2_O_2_	263.0821	263.0820	−0.38	235, 219, 132	Indirubin	P U B F
P21	10.50	C_27_H_21_N_3_O_3_	436.1661	436.1679	4.10	263, 160, 146	Indigolistin B2	P
P22	14.13	C_17_H_34_O_2_	271.2637	271.2638	0.37	135	n‐Heptadecanoic acid	U F
P23	14.20	C_10_H_16_	137.1330	137.1318	−8.70	121, 109, 95	α‐Pinene	P
P24	14.30	C_30_ H_48_O	425.3783	425.3773	−2.35	407, 283, 215	Lupenone	F

^a^Only characteristic fragment ions were shown.

^b^Indicate the mass error of molecular ions.

^c^P, plasma samples, U, urine samples, B, bile samples, and F, fecal samples.

**TABLE 2:** Metabolites (M1–M37) identified in rat biological samples after oral administration of indigo naturalis.

No.	RT (min)	Formula	[M + H]^+^ calculated (Da)^a^	[M + H]^+^ observed (Da)	Mass error^b^ (ppm)	Fragment ions	Identification	Matrix^c^
M1	0.98	C_7_H_7_NO_3_	154.0504	154.0507	2.00	138, 122	Hydroxyl‐2‐amino‐benzoic acid	P
M2	1.59	C_9_H_9_NO_5_S	244.028	244.0272	−3.28	164, 148, 120	1‐Methyl‐isatin‐3‐*O*‐sulfate	P U B
M3	2.10	C_18_H_22_N_2_O_8_	395.1454	395.1436	−4.56	219, 195	Hydrate‐methyl‐deoxyvasicinone glucuronide	U
M4	2.43	C_16_H_10_N_2_O_6_S	359.0338	359.0340	0.56	279, 263, 235	Indigo‐1‐*N*‐sulfate	U
M5	2.61	C_11_H_10_N_2_O_2_	203.0821	203.0815	2.95	185, 130	9‐Hydroxyl‐deoxyvasicinone	U
M6	3.51	C_8_H_8_N_2_O_3_	181.0613	181.0607	−3.31	147, 130, 120	1,3‐Dihydroxy‐2,3‐dihydroquinazolin‐4 (1H)‐one	U
M7	3.68	C_11_H_8_N_2_O_4_	233.0562	233.0575	5.60	187, 159	6,9,11‐Trihydroxyl‐deoxyvasicinone	P
M8	3.96	C_9_H_7_NO_2_	162.0555	162.0550	−3.09	148, 120, 92	1‐Methyl‐isatin	U
M9	4.09	C_7_H_5_NO	120.0449	120.0453	3.20	106, 92	Benzoazetinone	P
M10	4.24	C_11_H_10_N_2_O_3_	219.077	219.0769	−0.46	185, 130	9,11‐Dihydroxyl‐deoxyvasicinone	U
M11	4.25	C_22_H_18_N_2_O_10_	471.1040	471.1027	−2.76	295, 263	1‐Hydroxyl‐isoindigo‐1′‐*O*‐glucuronide	U
M12	4.38	C_28_H_28_N_2_O_14_	617.1619	617.1617	−0.32	263, 247, 130	Reduced‐indigo‐1,1′‐*N,N*‐diglucuronide	U
M13	4.58	C_28_H_26_N_2_O_14_	615.1462	615.1444	−2.92	439, 263	Indigo‐1,1′‐*N,N*‐diglucuronide	U
M14	4.79	C_22_H_18_N_2_O_8_	439.1141	439.1136	−1.14	263, 235, 219	Indigo‐4′‐*O*‐glucuronide	U
M15	4.93	C_22_H_18_N_2_O_8_	439.1141	439.1131	−2.28	263, 235, 219	Indigo‐1‐*N*‐glucuronide	U
M16	5.37	C_22_H_18_N_2_O_9_	455.1091	455.1082	1.98	279, 263, 235	Isoindigo‐1‐*O*‐glucuronide	P U
M17	5.62	C_15_H_10_N_2_O_3_	267.0770	267.0767	−1.12	249, 203	Hydrate‐tryptanthrin	U B
M18	6.02	C_16_H_10_N_2_O_4_	295.0719	295.0720	0.34	267, 263, 251	1,5‐Dihydroxyl‐indirubin	U
M19	6.42	C_16_H_10_N_2_O_4_	295.0719	295.0707	−4.07	279, 263, 235	1‐1′‐*N,N*‐Dihydroxyl‐indirubin	P U F
M20	6.44	C_22_H_18_N_2_O_9_	455.1091	455.1076	−3.29	279, 263, 249	2‐Methyl‐tryptanthrin‐8‐*O*‐glucuronide	U
M21	7.01	C_16_H_10_N_2_O_3_	279.0770	279.0773	1.10	265, 237, 221	Hydroxyl‐methyl‐tryptanthrin	P
M22	7.13	C_15_H_8_N_2_O_3_	265.0613	265.0622	3.39	237, 209	8‐Hydroxyl‐tryptanthrin	U
M23	7.22	C_16_H_10_N_2_O_3_	279.0770	279.0764	−2.15	263, 235, 219	1‐Hydroxyl‐indigo	U F
M24	7.24	C_22_H_20_N_2_O_7_	425.1349	425.1352	0.71	249, 235, 132	Decarbonyl‐methyl‐isoindigo glucuronide	P U
M25	7.34	C_22_H_18_N_2_O_8_	439.1141	439.1134	−1.60	263, 235	Indirubin‐1‐*N*‐glucuronide	P
M26	7.86	C_17_H_14_N_2_O_4_	311.1032	311.1031	−0.20	263, 201, 161	Trihydroxyl‐demethyl‐halichrome A	P
M27	8.08	C_16_H_10_N_2_O_3_	279.077	279.0761	−3.22	249, 235, 221	Hydroxyl‐methyl‐tryptanthrin	U F
M28	9.81	C_17_H_12_N_2_O_3_	293.0886	293.0898	4.09	279, 263, 235	1′‐*N,N*‐Hydroxyl‐1‐methyl‐isoindigo	P U
M29	9.91	C_17_H_12_N_2_O_4_	309.0875	309.0883	−2.59	263, 235, 219	1‐Hydroxyl‐1′‐methoxyl‐indigo	U F
M30	9.91	C_16_H_12_N_2_O_3_	281.0926	281.0925	−0.36	263, 235	Hydrate‐indigo	U F
M31	10.29	C_16_H_10_N_2_O_2_	263.0821	263.0821	0.00	249, 221	2‐Methyl‐tryptanthrin	U B
M32	10.40	C_17_H_12_N_2_O_2_	277.0937	277.0955	6.40	263, 235	Meisoindigo	P U B
M33	11.40	C_10_H_14_	135.1174	135.1168	−4.40	121, 105, 83	Desaturation‐α‐pinene	P
M34	11.58	C_42_H_66_O_14_	795.4531	795.4504	−3.39	443, 407	Hydrate‐lupenone diglucuronide	U
M35	11.90	C_8_H_6_O_2_	135.0446	135.0441	−3.70	120, 92	Benzofuran‐3‐ol	P
M36	14.20	C_10_ H_16_O	153.1279	153.1278	−0.80	137, 120	Hydroxyl‐α‐pinene	P
M37	14.30	C_10_H_14_	135.1174	135.1180	4.40	121, 105, 93, 83	Desaturation‐α‐pinene	P

^a^Only characteristic fragment ions were shown.

^b^Indicate the mass error of molecular ions.

^c^P, plasma samples, U, urine samples, B, bile samples, and F, fecal samples.

### 3.1. Identification of Prototypes in Rat Biological Samples (P1–P24)

Blank plasma, bile, urine, and feces samples collected from control rats (administered 0.5% sodium carboxymethylcellulose solution without IN) were analyzed under identical conditions. No peaks corresponding to the retention times of the 24 prototype compounds or 37 metabolites were detected in any of the blank matrices, confirming that the identified components were specifically derived from IN administration.

Investigation of detailed prototype fragmentation pathways is essential, as metabolite mass spectrometric fragmentation patterns often mirror those of their prototype compounds. Comparative analysis of metabolite product ion spectra with corresponding parent drugs facilitates structural speculation of metabolites. Additionally, certain product ions aid in predicting metabolite structures. The fundamental skeleton of prototype compounds typically remains intact in both phase I and phase II metabolites. This analytical process was supported by Mass Fragment software.

Utilizing EIC functionality, 24 prototype components were identified in drug‐containing rat biological samples following oral administration of IN. Proposed fragmentation pathways for selected prototypes are illustrated in Figure 1. For instance, three prototypes P13, P18, and P20 (RT = 6.17 min, 9.33 min, and 9.95 min) exhibited identical protonated molecular ions at m/z 263.08, with elemental composition corresponding to C16H10N2O2. Fragment ions at m/z 235 and m/z 219 indicated sequential losses of 28 Da (CO) and 44 Da (CO2) from the molecular ion. P18 and P20 were identified as indigo and indirubin, respectively, through comparison of retention times and accurate mass spectra with authentic standards. P13 was characterized as isoindigo based on chromatographic behavior comparison with literature data [29]. However, relative abundance ratios between fragment ions at m/z 235 and m/z 219 demonstrated significant variation among indigo, isoindigo, and indirubin in MS^2^ spectra. Compared to m/z 219 fragment abundance, m/z 235 fragment abundance was substantially lower for indirubin, comparable for indigo, and markedly higher for isoindigo. These fragmentation characteristics assisted in identifying related metabolites of indigo, isoindigo, and indirubin. Another prototype P16 (RT = 8.40 min) displayed an [M + H]+ ion at m/z 249.0658, with the speculated chemical formula C15H8N2O2 based on MassLynx elemental composition analysis. Fragment ions at m/z 221.07 and m/z 192.06 corresponded to [M + H‐CO]+ and [M + H‐2CO]+, respectively, leading to the identification of P16 as tryptanthrin through comparison of retention times and mass spectra with authentic standards. Blank control samples showed no peaks at corresponding retention times, confirming prototype presence in drug‐containing biological samples. Additional prototype compounds, including indole‐3‐aldehyde, 2‐methylquinazolin‐4(3H)‐one, quinazoline‐2,4(1H,3H)‐dione, 4(3H)‐quinazolinone, isatin, 2‐aminobenzoic acid, deoxyvasicinone, indoxyl, indole‐3‐acetic acid, isololiolide, 5,7,4′‐trihydroxy methoxyflavone, loliolide, phthalimide, phaitanthrin A, halichrome A, 7‐oxo‐7H‐benzopyrano[2,3‐c]cinnoline, indigolistin B2, n‐heptadecanoic acid, *α*‐pinene, and lupenone, were identified following analogous procedures and approaches.

### 3.2. Identification of Metabolites in Rat Biological Samples

Pharmaceutical compounds undergo both phase I and phase II metabolic pathways following oral administration. Oxidation, reduction, and hydrolysis represent major phase I metabolic processes, while phase II metabolic pathways involving N‐ or O‐glucuronidation and sulfonation constitute the primary metabolism of herbal medicines in vivo after oral intake. Glucuronide and sulfate conjugates exhibit characteristic mass fragments in metabolite identification, as their MS^2^ spectra typically display losses of 176 Da (C6H8O6) and 80 Da (SO3), respectively. Subsequently, aglycone fragment ions facilitate prototype identification. Isomeric compounds inevitably exist in IN extract and rat biological samples post‐administration. Calculated 1‐octanol/water partition coefficient (CLog P) values served as important parameters for metabolite identification, as compounds with higher CLog *P* values typically elute later during reversed‐phase chromatographic separation. For example, M14 and M15 with identical protonated ions at m/z 439.1141 (C22H18N2O8, RT = 4.79 min and 4.93 min, respectively) were 176 Da (C6H8O6) higher than the protonated molecular ion of indigo. Diagnostic loss of 176 Da corresponded to glucuronide conjugates, suggesting they represent glucuronidation metabolites of indigo (due to similar relative abundance of fragments at m/z 219 and m/z 235). Potential conjugation sites included 1N‐ or 4′O‐positions. M14 and M15 were tentatively identified as 4′O‐ and 1N‐glucuronidation products of indigo, respectively, although the exact conjugation positions remain hypothetical without NMR confirmation. Thirty‐seven metabolites were tentatively identified in rat biological samples following oral IN administration, structurally categorized into four groups, including indole alkaloid–related metabolites, quinazolinone alkaloid–related metabolites, pinene‐related metabolites, and other metabolites. Based on identified metabolites, potential in vivo metabolic pathways of active phytochemicals in IN were discussed.

#### 3.2.1. Indole Alkaloid–Related Metabolites (M2, M4, M8, M11–M16, M18, M19, M23–M26, M28–M30, and M32)

Indole alkaloids represent significant components within the alkaloid fraction of IN. Based on elemental composition and MS/MS fragmentation patterns, along with literature references, 19 indole alkaloid–related metabolites were tentatively identified, primarily derived from isatin, isoindigo, indigo, and indirubin. Proposed metabolic pathways are illustrated in Figure 3, mainly comprising N‐, O‐glucuronidation, N‐oxidation, N‐hydroxylation, dihydroxylation, and methylation, among others.

FIGURE 3The proposed metabolic pathways of indole alkaloid–related metabolites in rats.

**Figure:**
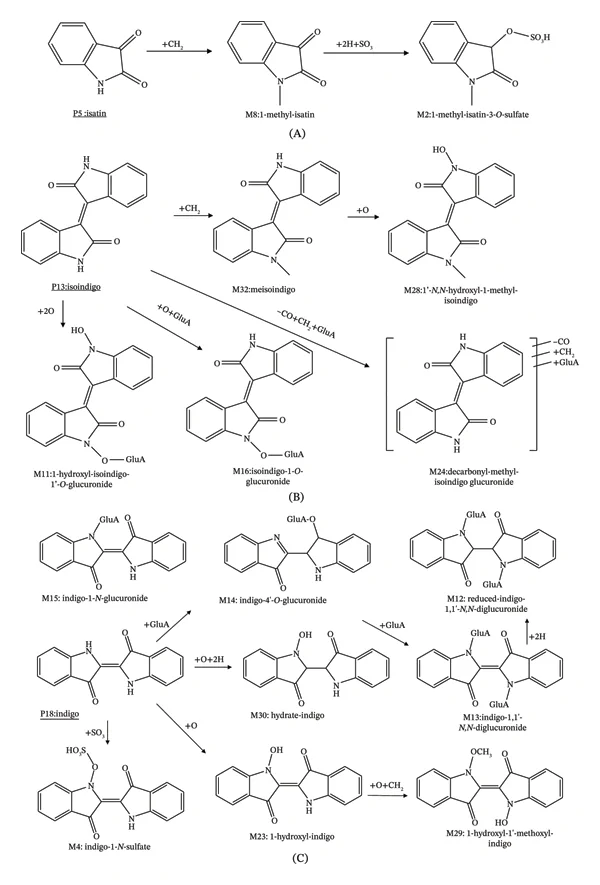

**Figure:**
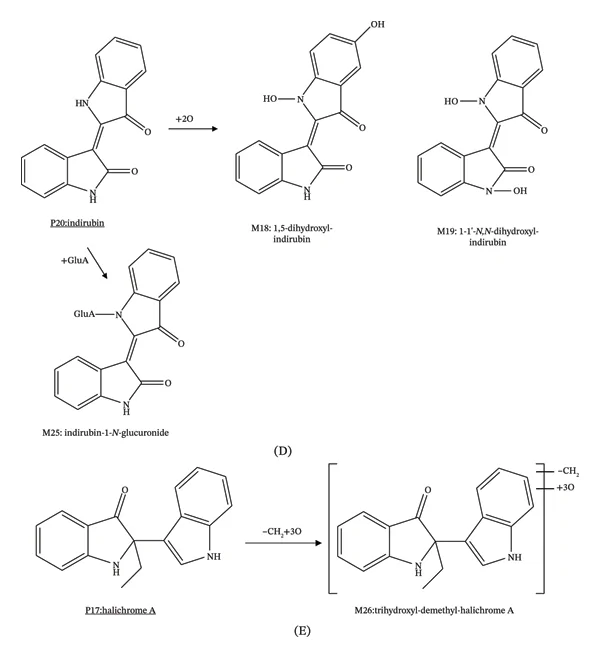

M15, M19, and M32 were selected as representative structures for dihydroxylated indole alkaloid–related metabolites, methylated indole alkaloid–related metabolites, and glucuronide‐conjugated indole alkaloid–related metabolites, respectively.

M19 (RT = 6.42 min) exhibited an [M+H]+ ion at m/z 295.0720, 32 Da (2O) higher than indirubin (m/z 263) in positive ion mode. Its chemical formula was deduced as C16H10N2O4 based on elemental composition, suggesting a dihydroxy metabolite of indirubin, indigo, or isoindigo. As shown in Figure 4, fragment ions at m/z 279 [M + H‐O]+, m/z 263 [M + H‐2O]+ indicated sequential 16 Da losses, suggesting the presence of two hydroxyl groups, and m/z 235 [M + H‐2O‐CO]+ indicated a subsequent CO loss. Since the relative abundance of m/z 235 was lower than m/z 219, resembling indirubin characteristics, we speculated M19 formation through dihydroxylation of indirubin. Previous literature [30] indicated the most probable conjugation site at N‐positions due to higher activity in dihydroxylated indole alkaloids. With only two nitrogen atoms in the skeleton, M19 was deduced to be 1,1′‐N,N‐dihydroxy‐indirubin.

**FIGURE 4:**
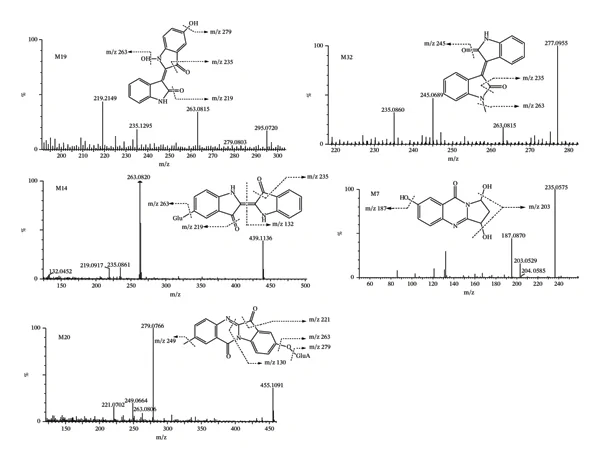
The product ion spectra and proposed fragmentation pathways of M14, M7, M19, M20, and M32 in positive ion mode.

M32 (RT = 10.40 min) displayed the [M + H]+ ion at m/z 277.0955, 14 Da (CH2) higher than indirubin (m/z 263) in positive ion mode. Its chemical formula was inferred as C16H10N2O4 from elemental composition, suggesting a methylation metabolite of isoindigo, indigo, or indirubin. As illustrated in Figure 4, fragment ions at m/z 263 [M + H‐CH2]+ indicated a 14 Da loss, suggesting methyl group presence, and m/z 235 [M + H‐CH2‐O]+ indicated subsequent hydroxyl loss. Since m/z 235 relative abundance substantially exceeded m/z 219, resembling isoindigo characteristics, we speculated M32 formation via the methylation metabolic pathway of isoindigo. The nitrogen atom was also hypothesized as the most probable conjugation position. Through literature review [22, 31, 32], M32 was identified as meisoindigo.

M15 was tentatively identified as indigo‐1‐N‐glucuronide. The proposed conjugation site is speculative and based on chromatographic behavior (CLog *p* values) and literature comparison. M15 with the protonated molecular ion at m/z 439.1141 (C22H18N2O8, RT = 4.79 min) was 176 Da (C6H8O6) higher than the protonated molecular ion of indigo. Diagnostic loss of 176 Da corresponded to glucuronide conjugates. Proposed fragmentation pathways for M15 are illustrated in Figure 4. Through MS behavior analysis compared to indigo, M15 was deduced to be indigo‐1‐N‐glucuronide.

#### 3.2.2. Quinazolinone Alkaloid–Related Metabolites (M3, M5–M7, M10, M17, M20, M21, M22, M27, and M31)

Quinazolinone alkaloids constitute another important component group within IN. Based on elemental composition and MS/MS fragmentation patterns, along with reference to related literature [25, 26], 12 quinazolinone alkaloid–related metabolites were tentatively identified, with proposed metabolic pathways including N‐, O‐glucuronidation, hydroxylation, dihydroxylation, trihydroxylation, and hydroxyl‐methylation. Proposed metabolic pathways are depicted in Figure 5.

**FIGURE 5:**
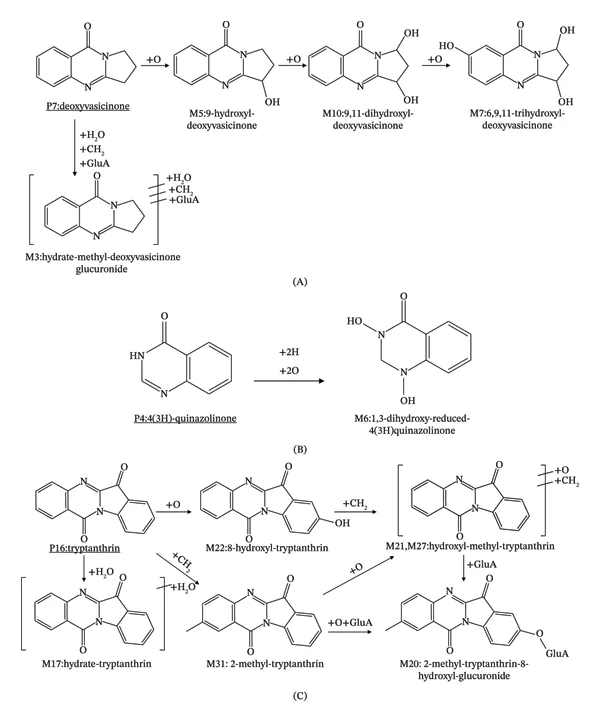
The proposed metabolic pathways of quinazolinone alkaloid–related metabolites in rats.

M5, M7, and M20 were selected as representative structures for hydroxylated quinazolinone alkaloid–related metabolites, trihydroxylated quinazolinone alkaloid–related metabolites, and glucuronide‐conjugated quinazolinone alkaloid–related metabolites, respectively.

M5 (RT = 2.61 min) exhibited the [M + H]+ ion at m/z 203.0815, 16 Da (O) higher than deoxyvasicinone (m/z 187) in positive ion mode. Its chemical formula was deduced as C11H10N2O2. A major fragment ion at m/z 185 indicated molecular H2O loss, and m/z 130 represented the characteristic mass fragment of deoxyvasicinone (P7) in positive ion mode. C‐9 was identified as the most plausible conjugation site [25]; therefore, M5 was deduced to be 9‐hydroxy‐deoxyvasicinone.

M7 (RT = 3.68 min) displayed the [M + H]+ ion at m/z 235.0575, 32 Da (2O) higher than M5 (m/z 203) in positive ion mode. Its chemical formula was inferred as C11H10N2O4, suggesting a dihydroxy metabolite of M5. As shown in Figure 5, fragment ions at m/z 203 [M + H‐2O]+ and m/z 187 [M + H‐3O]+ indicated sequential 16 Da losses, suggesting the presence of three hydroxyl groups. Its diagnostic mass fragment at m/z 203 resembled mass spectrometry characteristics of 9,11‐dihydroxy‐deoxyvasicinone, leading to speculation that hydroxyl group substitution sites were at C‐9 and 11 positions. An additional hydroxyl group might conjugate at the C‐6 position [25]; thus, M7 was deduced to be 6,9,11‐trihydroxy‐deoxyvasicinone.

M20 was tentatively identified as 2‐methyl‐tryptanthrin‐8‐O‐glucuronide, but the exact position of glucuronidation requires further confirmation. M20 with the protonated molecular ion at 455.1076 (C22H18N2O9, RT = 6.44 min) was 176 Da (C6H8O6) higher than the protonated molecular ion of M30. Diagnostic losses of 176 Da corresponded to glucuronide conjugates. As illustrated in Figure 5, major fragment ions at m/z 279 [M + H‐GluA]+, m/z 263 [M + H‐GluA‐CH2]+, m/z 249 [M + H‐GluA‐CH2‐O]+, and m/z 221 [M + H‐GluA‐CH2‐O‐CO]+ were observed in positive ion mode. Literature consultation [26, 31] revealed characteristic mass fragments similar to those of tryptanthrin, leading to identification of M20 as 2‐methyl‐tryptanthrin‐8‐O‐glucuronide.

#### 3.2.3. Terpenoid‐Related Metabolites (M33, M36, and M37)

Terpenoid compounds in IN demonstrate anti‐inflammatory, antifungal, and anti‐asthmatic effects, among others, closely associated with active *α*‐pinene content. In this investigation, based on elemental composition and MS/MS fragmentation patterns, along with reference to related literature, three *α*‐pinene–related metabolites were identified, with metabolic pathways primarily involving hydroxylation and desaturation. Their proposed metabolic pathways are illustrated in Figure 6.

**FIGURE 6:**
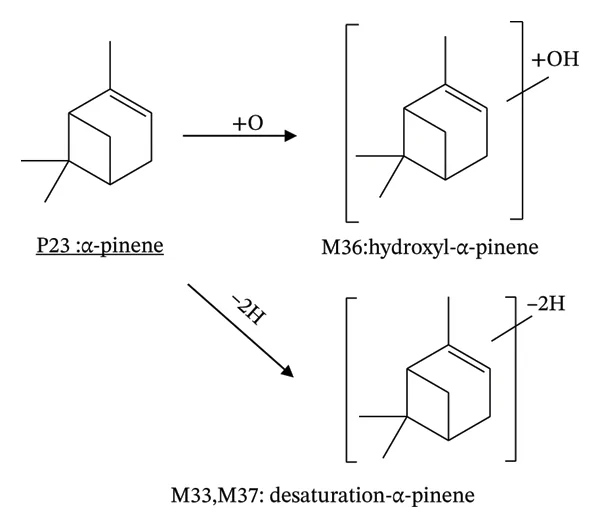
The proposed metabolic pathways of terpenoid‐related metabolites in rats.

M33 and M37 exhibited identical protonated molecular ions at m/z 135.1168 (C10H14, retention times 11.40 min and 14.30 min), representing 2 Da (2H) loss compared to the protonated molecular ion of *α*‐pinene (P23). Major fragment ions at m/z 121 [M + H‐CH2]+ were observed in positive ion mode. M33 and M37 were identified as desaturation metabolites of *α*‐pinene.

M36 (RT = 14.2 min) displayed the [M + H]+ ion at m/z 153.1278, 16 Da (O) higher than the protonated molecular ion of *α*‐pinene (m/z 137). The molecular formula was calculated as C10 H16O. Major fragment ions at m/z 137 [M + H‐O]+ indicated an additional hydroxyl group in the structure; thus, M36 was deduced to be hydroxy‐α‐pinene. The hydroxyl group may conjugate at the C‐4 position or methyl side chain, suggesting M36 could be myrtenol or verbenol [33].

#### 3.2.4. Other Metabolites (M1, M9, M34, and M35)

These metabolites do not represent major metabolic products and occur at low concentrations. Based on elemental composition and MS/MS fragmentation patterns, four additional metabolites were tentatively identified, with proposed major metabolic pathways illustrated in Figure 7.

**FIGURE 7:**
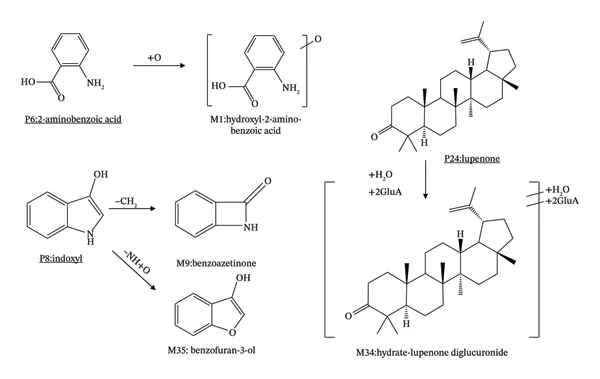
The proposed metabolic pathways of other metabolites in rats.

### 3.3. Analysis Results

Among the 61 identified components, four prototype compounds (indigo, indirubin, isoindigo, and tryptanthrin) were detected in all four biological matrices. Glucuronide conjugates (e.g., M14, M15, and M25) were exclusively observed in bile and urine but not in feces, suggesting complete deglucuronidation by intestinal bacteria before fecal excretion. Sulfate conjugates (e.g., M2 and M4) were primarily detected in urine. Notably, only phase I metabolites (e.g., M23, M27, M29, and M30) were found in feces, indicating that phase II conjugates are hydrolyzed in the intestinal tract. Detailed matrix distribution is provided in Table 1.

The UPLC–Q‐TOF MS method enabled rapid analysis of IN metabolic profiles in rats [34]. Compared to traditional detection methods, UPLC–Q‐TOF MS not only reduced analysis time but also provided enhanced detection sensitivity, facilitating identification of both major and minor metabolites. Following oral IN administration, 24 prototype compounds and 37 metabolites were identified in rat plasma, bile, urine, and feces samples. Results indicated that isoindigo, indigo, indirubin, tryptanthrin, and their related metabolites constitute the primary metabolic forms of IN *in vivo*.

Usami [21] identified reduced metabolites of isatin in human urine, which were not detected in our investigation, suggesting differences in reductase enzymes between humans and rats. Peng [22] observed that meisoindigo undergoes hydroxylation metabolic pathways following liver microsome incubation, with conjugation sites at C‐5, C‐6, and C‐7 positions. Wu and colleagues [23] investigated indirubin biosynthesis, discovering that N‐position methylation of indigo and indirubin enhances anticancer activity, establishing the N‐position as an active site of indirubin. Yang [24] documented that indirubin undergoes hydration, reduction, methylation, hydroxylation, disulfation, glucuronidation, and glutathionation metabolic pathways. Our study identified similar metabolic pathways except for glutathionation and disulfation. Plugar [25] examined deoxyvasicinone metabolism in rat urine, identifying two hydroxylated metabolites with confirmed hydroxylation positions. Our investigation additionally detected hydrated, methyl, and glucuronide metabolites of deoxyvasicinone. Jahng [26] studied tryptanthrin metabolism with rat liver microsomes, identifying hydroxylated and dihydroxylated metabolites. Our research represents the first detection of methylation and glutathionation metabolites, reflecting the increased complexity of in vivo biotransformation compared to in vitro systems. In summary, urine serves as the primary excretion route for IN following oral administration, containing more diverse and abundant metabolites.

Certain metabolites, including 2‐amino‐hydroxy‐benzoic acid, hydroxy‐methyl‐tryptanthrin, and hydroxy‐indirubin, were exclusively detected in plasma rather than urine, leading to preliminary speculation that these metabolites may undergo further metabolic processing in the kidneys.

(1H,3H)‐quinazoline‐2,4‐dione, tryptanthrin, indigo, and indirubin prototypes along with their related metabolites (including hydration, methylation, reduction, and sulfation) were identified in rat bile samples. Feces samples contained only phase I metabolites, with detected prototypes tryptanthrin, isoindigo, indigo, indirubin, n‐heptadecanoic acid, and lupenone alongside their related metabolites (including hydration, methylation, and hydroxylation). We postulate that certain IN components undergo extensive first‐pass metabolism by intestinal bacteria, gut epithelium, or liver following oral administration and absorption, subsequently undergoing conjugation with glucuronic acid in intestinal epithelium and liver via UGT enzymes, resulting in conjugate excretion through bile, urine, and feces. Although some conjugates may be excreted into the gut via bile, no conjugates were detected in feces, suggesting complete degradation of these conjugates by intestinal bacteria prior to excretion.

Our identification of tryptanthrin and its extensive phase II metabolites (M16, M17, M20, M22, and M31) in rat plasma, bile, and urine is consistent with the human pharmacokinetic data reported by Tang et al. [27], who found that tryptanthrin was rapidly absorbed and contributed significantly to systemic AhR activity. Interestingly, while Tang et al. [27] reported that indigo was not significantly elevated in human plasma post‐ingestion, we detected indigo (P18) and its glucuronide conjugates (M14 and M15) in rat plasma, suggesting potential species differences in the absorption and metabolism of indigo. Using a target constituent knock‐out strategy, Huang et al. [35] demonstrated that IN contains antileukemic components beyond indirubin and tryptanthrin, which aligns with our identification of 24 prototype compounds in rat biofluids, including less abundant indole alkaloids and terpenoids.

## 4. Conclusions

This investigation employed UPLC–Q‐TOF MS methodology combined with automated data analysis software (MetaboLynx) for rapid characterization of IN metabolic profiles in rat plasma, bile, urine, and feces following single‐dose administration. Overall, 24 prototype compounds and 37 metabolites were identified in rats, with 6 compounds confirmed through comparison with authentic standards and the remainder tentatively identified based on mass spectral fragmentation patterns, elution order, or literature references. Isoindigo, indigo, indirubin, tryptanthrin, and their related metabolites represent the primary metabolic forms of IN *in vivo*. Concurrently, metabolic pathways for IN extract and relationships between prototypes and their metabolites were proposed. To our knowledge, this represents the first comprehensive report on in vivo metabolic profiling of IN, providing scientific evidence for understanding potential metabolic pathways of major IN constituents.

It should be acknowledged that the majority of metabolites were tentatively identified based on accurate mass measurements, MS/MS fragmentation patterns, chromatographic elution order, and comparison with literature data, without confirmation using authentic standards. Future studies employing synthetic standards or NMR analysis are needed to fully validate the proposed structures.

Our findings, together with the human pharmacokinetic data from Tang et al. [27], provide complementary evidence that tryptanthrin and its metabolites warrant further investigation as key contributors to the systemic effects of IN.

## Author Contributions

Guozhe Zhang and Jianting Liu contributed to the writing–original draft, software, and data curation. Dan Yuan and Yiyu Qin contributed to the writing–review and editing.

## Funding

This study was supported by the Jiangsu Provincial Higher Education Natural Science Foundation, 19KJB350010; Qinyu detergent in‐house formulation development project, 20238112.

## Disclosure

All authors approved the final version of the manuscript.

## Conflicts of Interest

The authors declare no conflicts of interest.

## Supporting Information

Additional supporting information can be found online in the Supporting Information section.

## Supporting information


**Supporting Information** Table S1 lists all compounds identified in indigo naturalis by UPLC–Q‐TOF MS, including retention time, molecular formula, calculated and observed [M+H]+ m/z, mass error (ppm), and MS/MS fragment ions.

## Data Availability

The data used to support the findings of this study are included within the manuscript and are available from the corresponding author upon request.

## References

[1] Sun X. L. , Tang L. W. , Zhang L. Q. , and Fu C. H. , A Comprehensive Review of Indigo Naturalis: Chemical Constituents, Pharmacokinetics, Pharmacology, Clinical Applications, Adverse Events, and Quality Control, Frontiers in Pharmacology. (2021) 12. 10.3389/fphar.2021.664022 PMC8200773 34135755

[2] Xie J. , Tian S. , Liu J. et al., Combination Therapy With Indigo and Indirubin for Ulcerative Colitis via Reinforcing Intestinal Barrier Function, Oxidative Medicine and Cellular Longevity. (2023) 2023, 1–24, 10.1155/2023/2894695. PMC9943625 36825081

[3] Tu P. , Tian R. , Lu Y. et al., Beneficial Effect of Indigo Naturalis on Acute Lung Injury Induced by Influenza A Virus, Chinese Medicine. (2020) 15, no. 1, 10.1186/s13020-020-00415-w. PMC7750395 33349263

[4] Yang X. , Tang J. , Su J. et al., High-Quality Indigo Naturalis Obtained With Automatic Foam Separation, ACS Applied Materials & Interfaces. (2023) 15, no. 36, 43272–43281, 10.1021/acsami.3c04112. 37669429

[5] Yin-Ku L. , Andres P. , and Chantalat L. , Antibacterial Indigo Naturalis or Indigo-Producing Plant Extract and Use Thereof, 2018, Google Patents.

[6] Chen S. , Qin W. , Lu X. et al., Arsenic Trioxide Versus Realgar-Indigo Naturalis Formula in Non-High-Risk Acute Promyelocytic Leukemia: A Multicenter, Randomized Trial, Haematologica. (2025) 110, no. 2, 621–628, 10.3324/haematol.2024.285905. PMC11873696 39506905

[7] Ginzinger W. , Egger A. , Muhlgassner G. et al., Water-Soluble Cationic Derivatives of Indirubin, the Active Anticancer Component From Indigo Naturalis, Chemistry and Biodiversity. (2012) 9, no. 10, 2175–2185, 10.1002/cbdv.201200147. 23081917

[8] Liu C. , Zhang X.-M. , Liu L.-J. et al., Analysis of Mechanism of Indigo Naturalis in Treating Chronic Myelocytic Leukemia Based on Two-Dimensional Model of Protein-Protein Interaction Network--Molecular Docking Technique, TMR Theory Hypothesis. (2018) 1, no. 2, 13–17.

[9] Wu D. , Huang Q. , Xu Y. et al., Clinical Efficacy and Future Application of Indigo Naturalis in the Treatment of Ulcerative Colitis, Journal of Ethnopharmacology. (2025) 337, 10.1016/j.jep.2024.118782. 39236777

[10] Kondo S. , Araki T. , Okita Y. et al., Colitis With Wall Thickening and Edematous Changes During Oral Administration of the Powdered Form of Qing-Dai in Patients With Ulcerative Colitis: A Report of Two Cases, Clinical Journal of Gastroenterology. (2018) 11, no. 4, 268–272, 10.1007/s12328-018-0851-7. 29549501

[11] Urushikubo J. , Yanai S. , Nakamura S. et al., Efficacy of Indigo Naturalis Therapy for Ulcerative Colitis: A Case Series, Internal Medicine. (2019) 58, no. 16, 2299–2304, 10.2169/internalmedicine.2446-18. PMC6746630 31118381

[12] Naganuma M. , Treatment With Indigo Naturalis for Inflammatory Bowel Disease and Other Immune Diseases, Immunological Medicine. (2019) 42, no. 1, 16–19, 10.1080/25785826.2019.1599158. 31034341

[13] Damevska K. , França K. , Lotti T. , Nikolovska S. , Pollozhani N. et al., Complementary and Integrative Therapies for Psoriasis: Looking Forward, Dermatologic Therapy. (2018) 31, no. 6, 10.1111/dth.12627. 30133906

[14] Gaitanis G. , Magiatis P. , Velegraki A. , Bassukas I. D. et al., A Traditional Chinese Remedy Points to a Natural Skin Habitat: Indirubin (Indigo Naturalis) for Psoriasis and the Malassezia Metabolome, British Journal of Dermatology. (2018) 179, no. 3, 10.1111/bjd.16807. 29791716

[15] Farahnik B. , Sharma D. , Alban J. , Sivamani R. K. et al., Topical Botanical Agents for the Treatment of Psoriasis: A Systematic Review, American Journal of Clinical Dermatology. (2017) 18, no. 4, 451–468, 10.1007/s40257-017-0266-0. 28289986

[16] Kim M. H. , Choi Y. Y. , Yang G. et al., Indirubin, a Purple 3,2-bisindole, Inhibited Allergic Contact Dermatitis via Regulating T Helper (Th)-Mediated Immune System in DNCB-Induced Model, Journal of Ethnopharmacology. (2013) 145, no. 1, 214–219, 10.1016/j.jep.2012.10.055. 23149289

[17] Naganuma M. , Sugimoto S. , Suzuki H. et al., Adverse Events in Patients With Ulcerative Colitis Treated With Indigo Naturalis: A Japanese Nationwide Survey, Journal of Gastroenterology. (2019) 54, no. 1, 1–6, 10.1007/s00535-019-01591-9. 31102012

[18] Matsuno Y. , Hirano A. , and Esaki M. , Possible Association of Phlebitis-Induced Colitis With Indigo Naturalis, Gastroenterology. (2018) 155, no. 3, 576–577, 10.1053/j.gastro.2018.01.074. 30064721

[19] Naganuma M. , Sugimoto S. , Mitsuyama K. et al., Efficacy of Indigo Naturalis in a Multicenter Randomized Controlled Trial of Patients With Ulcerative Colitis, Gastroenterology. (2018) 154, no. 4, 935–947, 10.1053/j.gastro.2017.11.024. 29174928

[20] Adachi S. , Hoshi N. , Inoue J. et al., Indigo Naturalis Ameliorates Oxazolone-Induced Dermatitis but Aggravates Colitis by Changing the Composition of Gut Microflora, International Archives of Allergy and Immunology. (2017) 173, no. 1, 23–33, 10.1159/000471923. 28482341

[21] Usami N. , Kitahara K. , Ishikura S. , Nagano M. , Sakai S. , and Hara A. , Characterization of a Major Form of Human Isatin Reductase and the Reduced Metabolite, European Journal of Biochemistry. (2001) 268, no. 21, 5755–5763, 10.1046/j.0014-2956.2001.02510.x. 11722560

[22] Peng Y. , Study on the Metabolic Transformation of the Anticancer Drug Isoindigo, 1989, China Union Medical University.

[23] Wu K. M. , Zhang M. Y. , Fang Z. , and Huang L. , Synthesis of Antileukemic Drugs Indirubin, Indigo, Isoindigo and Their Derivatives, Acta Pharmaceutica Sinica. (1985) 11, no. 11, 821–826. 3869774

[24] Yang B. , Studies on Metabolism of Indirubin in Vivo and in Vitro, 2009, Third Military Medical University, Chongqing, China.

[25] Abdullaev N. , Rashkes Y. V. , Yagudaev M. , Tulyaganov N. et al., Structure of the Products of the Metabolism of Deoxypeganine and of Deoxyvasicinone, Chemistry of Natural Compounds. (1983) 19, no. 6, 720–727.

[26] Jahng Y. , Progress in the Studies on Tryptanthrin, an Alkaloid of History, Archives of Pharmacal Research. (2013) 36, no. 5, 517–535, 10.1007/s12272-013-0091-9. 23543631

[27] Tang J. S. , Cait A. , O’Sullivan D. et al., Pharmacokinetics of Major Indigo Naturalis Indole Alkaloids in Humans and Their Association With Systemic Aryl Hydrocarbon Receptor Activity, Biochemical Pharmacology. (2025) 235. 10.1016/j.bcp.2025.116805 39993611

[28] Zhang G. , Sun J. , Kano Y. , and Yuan D. , Metabolic Profile of Irisolidone in Rats Obtained by Ultra-High Performance Liquid Chromatography/Quadrupole Time-of-Flight Mass Spectrometry, Journal of Analytical Methods in Chemistry. (2013) 2013, no. 941, 1–9, 10.1016/j.jchromb.2013.09.033. 24184829

[29] Liau B. C. , Jong T. T. , Lee M. R. , and Chen S. S. , LC-APCI-MS Method for Detection and Analysis of Tryptanthrin, Indigo, and Indirubin in Daqingye and Banlangen, Journal of Pharmaceutical and Biomedical Analysis. (2007) 43, no. 1, 346–351, 10.1016/j.jpba.2006.06.029. PMC7126482 16884885

[30] Chiu S. H. L. and Huskey S. W. , Species Differences in N-Glucuronidation, Drug Metabolism and Disposition. (1998) 26, no. 9, 838–847. 9733661

[31] Yang X. W. , Metabolic Analysis of TCM Components, 2003, China Medical Science Press, Beijing, China.

[32] Peng Y. , Study on Metabolic Biotransformation of Anticancer Drug Meisoindigo, Ph.D. Dissertation. (1989) China Union Medical College, Beijing, China.

[33] Schmidt L. and Göen T. , Human Metabolism of α-Pinene and Metabolite Kinetics After Oral Administration, Archives of Toxicology. (2017) 91, no. 2, 677–687, 10.1007/s00204-015-1656-9. 26679931

[34] Zhang G. , Xiao L. , and Qi L. , Metabolite Profiling of Meridianin C in Vivo of Rat by UHPLC/Q-TOF MS, Journal of Analytical Methods in Chemistry. (2021) 2021, 1–10, 10.1155/2021/1382421. PMC8553504 34721922

[35] Huang H. , Li Y. , Dai Y. et al., Antileukemic Effects of Indigo Naturalis Constituents by ’Target Constituent Knock-Out’ Coupled With Semipreparative Liquid Chromatography and Quadrupole Time-of-Flight Mass Spectrometry, Biomedical Chromatography. (2021) 35, no. 12, 10.1002/bmc.5216. 34254701

