# Supplementary material for: Metabolic Profile of Indigo Naturalis in Rat Plasma, Bile, Urine, and Feces by UPLC–Q‐TOF MS Method
Source: J Anal Methods Chem. 2026 Oct 27;2026:8592761. doi: 10.1155/jamc/8592761 (PMC13711370; doi:10.1155/jamc/8592761)
Supplement: Supplementary file 1 [file JAMC-2026-8592761-s001.doc]

#### Table S1 The compounds found from Indigo Naturalis by UPLC-Q-TOF MS method

| No. | RT  (min) | Formula | [M+H]^+^ (*m/z*) | | | MS/MS | | Identification |
| --- | --- | --- | --- | --- | --- | --- | --- | --- |
|  |  |  | Calculated | Observed | Error  (ppm) |  |  |  |
| 1 | 0.60 | C_6_H_13_NO_2_ | 132.1025 | 132.1021 | -3.03 | | 118, 88 | isoleucine |
| 2 | 0.63 | C_5_H_11_NO_2_ | 118.0868 | 118.0873 | 4.23 | | 102, 70 | valine |
| 3 | 1.08 | C_6_H_13_NO_2_ | 132.1025 | 132.1025 | 0.00 | | 118, 88 | leucine |
| 4 | 1.38 | C_8_H_6_N_2_O | 147.0558 | 147.0562 | 2.72 | | 132, 129, 119, 102, 92 | 2H-cinnolin-3-one |
| 5 | 2.16 | C_8_H_7_NO | 134.0606 | 134.0616 | 7.46 | | 116, 106, 92 | 5,6-dihydrocyclopenta[b]pyridin-7-one |
| 6 | 2.25 | C_12_H_22_N_2_O_2_ | 227.1760 | 227.1757 | -1.32 | | 209, 133, 123 | N-[(1R,2R)-2-[acetyl(methyl)amino]cyclohexyl]-N-methylacetamide |
| 7 | 2.48 | C_9_H_7_NO | 146.0606 | 146.0611 | 3.42 | | 117, 92 | indole-3-aldehyde |
| 8 | 2.79 | C_9_H_8_N_2_O | 161.0715 | 161.0714 | -0.62 | | 146, 130, 120 | 2-methylquinazolin-4(3H)-one |
| 9 | 3.18 | C_8_H_6_N_2_O_2_ | 163.0508 | 163.0507 | -0.61 | | 146, 120, 90 | (1H,3H)-quinazoline-2,4-dione |
| 10 | 3.22 | C_8_H_6_N_2_O | 147.0558 | 147.0558 | 0.00 | | 130, 120, 90 | 4(3H)-quinazolinone |
| 11 | 3.81 | C_8_H_5_NO_2_ | 148.0399 | 148.0397 | -1.35 | | 130, 120, 92, 79 | isatin |
| 12 | 4.18 | C_7_H_7_NO_2_ | 138.0555 | 138.0559 | 2.90 | | 120, 92 | 2-aminobenzoic acid |
| 13 | 4.33 | C_11_H_10_N_2_O | 187.0871 | 187.0873 | 1.07 | | 147, 130, 120 | deoxyvasicinone |
| 14 | 4.37 | C_8_H_7_NO | 134.0606 | 134.0603 | -2.24 | | 117, 79 | indoxyl |
| 15 | 4.62 | C_10_H_9_NO_2_ | 176.0712 | 176.0699 | -7.38 | | 159, 120, 92 | indole-3-acetic acid |
| 16 | 4.81 | C_11_H_16_O_3_ | 197.1178 | 197.1161 | -8.62 | | 183, 179, 147, 135 | isololiolide |
| 17 | 4.99 | C_16_H_12_O_6_ | 301.0712 | 301.0699 | -4.32 | | 286, 152, 149 | 5,7,4'-trihydroxy methoxyflavone |
| 18 | 5.22 | C_11_H_16_O_3_ | 197.1178 | 197.1164 | -7.10 | | 183, 179, 147, 135 | loliolide |
| 19 | 5.26 | C_15_H_10_N_2_ | 219.0922 | 219.0916 | -2.74 | | 130, 118, 91 | 10H-indolo,[3,2-b],quinoline |
| 20 | 5.45 | C_12_H_10_N_4_O_2_ | 243.0882 | 243.0866 | -6.58 | | 133, 120, 107, 105 | lumichrome |
| 21 | 5.87 | C_11_H_11_NO_3_ | 206.0817 | 206.0827 | 4.85 | | 130, 120 | 3-(2-oxopropyl)-3-hydroxyindol-2-one |
| 22 | 5.9 | C_16_H_11_N_3_O | 262.0980 | 262.0979 | -0.38 | | 235, 219, 190, 120 | 7-aminobenzo[b][1,7]phenanthrolin-3(4H)-one |
| 23 | 6.16 | C_15_H_12_N_2_O_2_ | 253.0977 | 253.0987 | 3.95 | | 235, 162, 146, 134 | 3-(2'-hydroxyphenyl)-2-methyl-4(3H)-quinazolinone |
| 24 | 6.17 | C_16_H_10_N_2_O_2_ | 263.0821 | 263.0819 | -0.76 | | 235, 219, 190, 132 | isoindigo |
| 25 | 6.48 | C_15_H_10_N_2_O | 235.0871 | 235.0873 | 0.85 | | 219, 146, 120, 92 | quindolinone |
| 26 | 6.53 | C_8_H_5_NO_2_ | 148.0399 | 148.0393 | -4.05 | | 130, 120, 105 | Phthalimide |
| 27 | 6.87 | C_14_H_16_N_2_O_6_ | 309.1087 | 309.1064 | -7.44 | | 247, 201, 149 | Indicaxanthin |
| 28 | 6.97 | C_7_H_5_NO | 120.0449 | 120.0452 | 2.50 | | 92, 77 | 1,3-benzoxazole |
| 29 | 7.30 | C_8_H_6_N_2_O_2_ | 163.0508 | 163.0521 | 7.97 | | 147, 120 | 2-hydroxylquinazolin-4(3H)-one |
| 30 | 7.38 | C_18_H_14_N_2_O_3_ | 307.1083 | 307.1060 | -7.49 | | 289, 263, 261, 249 | phaitanthrin A |
| 31 | 7.98 | C_24_H_17_N_3_O_4_ | 412.1297 | 412.1328 | 7.52 | | 303, 275, 263 | indigolistin C |
| 32 | 8.02 | C_15_H_10_N_2_O_3_ | 267.0770 | 267.0774 | 1.50 | | 249, 221, 130, 120 | 3-(2'-carboxyphenyl)-4(3H)-quinazolinone |
| 33 | 8.40 | C_15_H_8_N_2_O_2_ | 249.0664 | 249.0664 | 0.00 | | 221, 130, 120, 92 | tryptanthrin^[18]^ |
| 34 | 8.59 | C_16_H_10_N_2_O_3_ | 279.0770 | 279.0751 | -6.81 | | 263, 235, 219 | hydroxyindirubin |
| 35 | 9.34 | C_18_H_16_N_2_O | 277.1341 | 277.1351 | 3.61 | | 248, 219, 160 | halichrome A |
| 36 | 9.36 | C_16_H_10_N_2_O_2_ | 263.0821 | 263.0815 | -2.28 | | 235, 219, 170, 132 | indigo |
| 37 | 9.88 | C_24_H_16_N_4_O | 377.1402 | 377.1380 | -5.83 | | 258, 256, 245, 120 | N-2-phenyl-5,7-dihydropyrido[3,2-b:5,6-b']-diindole-6-carboxamide |
| 38 | 9.89 | C_14_H_15_NO_7_ | 310.0927 | 310.0939 | 3.87 | | 134, 79 | indoxyl glucuronide |
| 39 | 9.91 | C_15_H_8_N_2_O_2_ | 249.0664 | 249.0662 | -0.80 | | 221, 192, 146, 130 | 7-oxo-7H-benzopyrano[2,3-c]cinnoline |
| 40 | 9.91 | C_23_H_13_N_3_O_2_ | 364.1086 | 364.1089 | 0.82 | | 335, 259, 230 | qingdainone |
| 41 | 9.94 | C_19_H_13_N_3_O | 300.1137 | 300.1131 | -2.00 | | 258 | 3-methyl-9-phenyl-9H-isoxazolo[4',5':5,6]pyrido[2,3-b]indole |
| 42 | 9.96 | C_16_H_10_N_2_O_2_ | 263.0821 | 263.0818 | -1.14 | | 235, 219, 190 | indirubin |
| 43 | 10.32 | C_27_H_21_N_3_O_3_ | 436.1661 | 436.1666 | 1.15 | | 263, 160, 146 | indigolistin B1 |
| 44 | 10.50 | C_27_H_21_N_3_O_3_ | 436.1661 | 436.1657 | -0.92 | | 263, 160, 146 | indigolistin B2 |
| 45 | 10.83 | C_27_H_21_N_3_O_3_ | 436.1661 | 436.1657 | -0.92 | | 263, 160, 146 | indigolistin B3 |
| 46 | 11.03 | C_28_H_23_N_3_O_3_ | 450.1818 | 450.1841 | 5.11 | | 273, 263, 160, 142 | indigolistin A |
| 47 | 11.84 | C_27_H_30_O_15_ | 595.1663 | 595.1639 | -4.03 | | 475, 433, 168, 118 | saponarin |
| 48 | 11.87 | C_7_H_10_ | 95.0861 | 95.0866 | 5.26 | | 69, 67 | norbornene |
| 49 | 12.71 | C_8_H_4_O_3_ | 149.0239 | 149.0242 | 2.01 | | 121, 93 | phthalic anhydride |
| 50 | 13.11 | C_32_H_18_N_4_O_2_ | 491.1508 | 491.1503 | -1.02 | | 463, 372, 116 | bisindigotin |
| 51 | 14.18 | C_17_H_34_O_2_ | 271.2637 | 271.2627 | -3.69 | | 191, 135, 125, 111 | n-heptadecanoic acid |
| 52 | 14.28 | C_30_H_48_O | 425.3783 | 425.3789 | 1.41 | | 407, 283, 215, 109 | lupenone |
| 53 | 14.30 | C_10_H_16_ | 137.1330 | 137.1335 | 3.65 | | 121, 109, 95 | (-)-α-pinene |
